# *Notes from the Field: Mycobacteria chimaera* Infections Associated with Heater-Cooler Unit Use During Cardiopulmonary Bypass Surgery — Los Angeles County, 2012–2016

**DOI:** 10.15585/mmwr.mm675152a4

**Published:** 2019-01-04

**Authors:** M. Claire Jarashow, Dawn Terashita, Sharon Balter, Benjamin Schwartz

**Affiliations:** ^1^Los Angeles County Department of Public Health, California; ^2^Division of Scientific Education and Professional Development, Center for Surveillance, Epidemiology and Laboratory Services, CDC.

In December 2016, hospital A in Los Angeles County, California, reported two *Mycobacterium avium* complex infections, later identified as *Mycobacterium chimaera*, in patients with a recent history (<5 years) of cardiopulmonary bypass surgery. Both surgical procedures used the Sorin Stöckert 3T (Sorin Group, Munich, Germany) heater-cooler unit brand (currently LivaNova PLC, London, United Kingdom) to heat and cool blood. These heater-cooler units have been linked to outbreaks of *M. chimaera* infections among patients with similar surgical histories in Europe and the United States (*1*,*2*). Sorin Stöckert 3T heater-cooler units contaminated during manufacturing before September 2014 were identified as the source of infection through emission of bioaerosols containing *M. chimaera* during surgery (*3*); these units have been removed and replaced by hospital A. 

*M. chimaera* is a nontuberculous mycobacterium first described in 2004 (*4*). *M. chimaera* infection diagnosis is challenging because clinical manifestations can take months or years to develop and are often nonspecific. Infections have been diagnosed up to 6 years after initial surgical exposure (*5*). Acid-fast bacillus cultures might not be ordered, or results might be negative given the slow-growing nature of *M. chimaera* (*5*,*6*). In hospitals with confirmed *M. chimaera* infections, reported incidence rates among heater-cooler unit–exposed patients ranged from one per 100 persons to one per 1,000 persons (*2*,*5*), and the case-fatality rate was approximately 50% (*6*,*7*). Infections were reported most frequently among patients who had valve replacement or other implants during surgery (*8*).

CDC released a health alert in October 2016 recommending that hospitals that used Sorin Stöckert 3T heater-cooler units notify patients who were potentially exposed during 2012–2016. Because hospital A used implicated heater-cooler units, an investigation was initiated by the Los Angeles County Department of Public Health in December 2016, to enhance case findings and implement control measures. During the investigation, approximately 4,000 patients were sent letters per CDC guidance, describing the potential exposure and instructing them to seek care if they experienced signs or symptoms consistent with *M. chimaera* infection, such as fatigue, unexplained fever, night sweats, weight loss, or wound infection. A nurse call center was established to answer patient questions and refer to care when necessary. All relevant clinical staff members were notified, and an alert was inserted into electronic health records of potentially exposed patients. Hospital A was advised to report all *M. chimaera* cases to the Food and Drug Administration via MedWatch.

By May 2017, 20 confirmed cases of *M. chimaera* infection had been identified, defined as isolation of culture-positive nontuberculous mycobacterium from an invasive nonpulmonary specimen, with *M. chimaera* species identification by DNA sequencing of 16S rRNA, in a patient with a history of cardiopulmonary bypass during 2013–2016. Fifteen (75%) cases were identified by clinicians during patient hospitalization, follow-up care, or subsequent surgical procedures at hospital A or affiliated facilities. Five (25%) patients sought care because they received a patient notification letter and subsequently received a diagnosis of *M. chimaera* infection. All five patients identified through patient notification letters had valve replacements or implants inserted during surgery, and all five remain alive. Thirteen of the 15 patients identified during hospitalization, follow-up care, or subsequent surgery had valve replacements or implants, and eight of these 15 patients were alive at the time this report was produced.

Informing and reminding exposed persons to seek care for *M. chimaera–*associated nonspecific symptoms can be important for diagnosis, particularly because subsequent care might not occur at the exposure hospital, limiting the likelihood of complete exposures being known. Because of *M. chimaera’s* long incubation time, hospitals that used implicated heater-cooler units could consider additional proactive steps toward early detection of infection, such as annual patient renotification and implementation of clinician alerts in electronic medical records.
